# Defining the Optimal Time of Adaptive Replanning in Prostate Cancer Patients with Weight Change during Volumetric Arc Radiotherapy: A Dosimetric and Mathematical Analysis Using the Gamma Index

**DOI:** 10.1155/2017/4149591

**Published:** 2017-12-18

**Authors:** Hoon Sik Choi, Guang Sub Jo, Jong Pyo Chae, Sang Bong Lee, Chul Hang Kim, Bae Kwon Jeong, Hojin Jeong, Yun Hee Lee, In Bong Ha, Ki Mun Kang, Jin Ho Song

**Affiliations:** ^1^Department of Radiation Oncology, Gyeongsang National University School of Medicine and Gyeongsang National University Changwon Hospital, Changwon 51472, Republic of Korea; ^2^Department of Radiation Oncology, Gyeongsang National University School of Medicine and Gyeongsang National University Hospital, Jinju 52727, Republic of Korea; ^3^Institute of Health Science, Gyeongsang National University, Jinju 52727, Republic of Korea

## Abstract

We evaluated the changes in the dose distribution of radiation during volumetric arc radiotherapy (VMAT), to determine the right time for adaptive replanning in prostate cancer patients with progressive weight (WT) changes. Five prostate cancer patients treated with VMAT were selected for dosimetric analysis. On the original computed tomography images, nine artificial body contours were created to reflect progressive WT changes. Combined with three different photon energies (6, 10, and 15-MV), 27 comparable virtual VMAT plans were created per patient. The dosimetric analysis included evaluation of target coverage (*D*_95%_, *D*_max_), conformity index, homogeneity index, and organs at risk doses. The dose differences among the plans were determined using the gamma index analysis and were compared with the dosimetric analysis. Mean *D*_95%_ became lower than 98% when body contour expanded by 2.0 cm or more and *D*_max_ became higher than 107% when body contour contracted by 1.5 cm or more in 10-MV plans. This cut-off values correlated well with gamma index analysis results. Adaptive replanning should, therefore, be considered if the depth of body contour becomes 1.5 cm smaller (WT loss) or 2.0 cm larger (WT gain) in patients treated by VMAT with 10-MV photons.

## 1. Introduction

The goal of radiotherapy (RT) is to maximize tumor control while minimizing damage to the surrounding normal tissue [1]. To achieve this goal, conventional RT techniques have been replaced with more sophisticated delivering methods, such as intensity-modulated radiotherapy (IMRT) and volumetric modulated arc radiotherapy (VMAT), which can create steeper dose gradients between the tumor and normal tissue [2–5]. However, if these techniques are not supported by image-guided radiotherapy (IGRT) which uses various in-room imaging devices, the steep dose gradient can increase the risk of missing the target [6, 7]. With the aid of IGRT techniques such as the in-room cone-beam computed tomography (CT), it is possible to study the set-up errors and the anatomical changes during the RT session. However, it is difficult to determine if the observed anatomical changes are significant enough to consider a new adaptive radiotherapy (ART) plan [8–10].

Prostate cancer is the most common cancer in men, and RT is often used for the treatment of this cancer [11, 12]. Due to the radiobiological features of prostate cancer, often higher doses of RT are required for long durations. However, since the rectum and bladder are in proximity to the prostate gland, a very high level of precise ART technique is essential [13, 14]. For ART, various anatomical changes must be considered, and a change in body weight (WT) is one of them. WT changes are often observed in prostate cancer patients due to various reasons such as dehydration and loss of appetite or as side effects of hormone therapy [15, 16]. Smith et al. [15] reported that body WT of patients receiving androgen deprivation increased by 2.4 ± 0.8%. These body WT and body contour changes could affect the radiation dose distribution. Chow and Jiang [16] reported that a 2 cm decrease in contour depth, caused by the patient WT loss, could increase the dose to the target and normal organs by more than 5%. Since WT changes in every patient vary, individualized ART is needed. However, there are no standard criteria for the optimal time to consider ART.

Therefore, in this study, we calculated and analyzed the dosimetric changes for the targets and organs at risk (OAR) as the patient's WT changed. With the addition of the gamma index, which is usually used to quantify the quality of the delivered calculated plans, we have suggested criteria which will help in deciding the optimal time to consider ART in prostate cancer patients with WT changes.

## 2. Materials and Methods

### 2.1. Patients and Radiotherapy

Five prostate cancer patients treated at the Gyeongsang National University Changwon Hospital between July 2016 and May 2017 were selected for the study. All patients were treated by VMAT due to low- to intermediate-risk (on the basis of the National Comprehensive Cancer Network) prostate cancer. All patients were immobilized in the supine position with an empty rectum and full bladder. CT images were taken and imported into the Eclipse treatment planning system Version 13.7 (Varian Inc., Sunnyvale, CA, USA). The targets and OAR were contoured on these CT images. The prostate and seminal vesicle were delineated as the gross tumor volume (GTV). The clinical target volume (CTV) was defined to be the same as the GTV. The planning target volume (PTV) was created by expanding the CTV by 5 mm except 3 mm posteriorly. The rectum and bladder were delineated as OAR. The VMAT plans were made using double 360-degree photon arcs with high definition 120-leaf multileaf collimator from the Varian TrueBeam (Varian Medical Systems, Palo Alto, CA, USA). All patients were prescribed a dose of 78 Gy in 39 fractions. The VMAT plan was normalized so that 95% of the PTV received more than 100% of the prescribed dose. Based on the QUANTEC (Quantitative Analyses of Normal Tissue Effects in the Clinic) guidelines radiation exposure of the rectum was limited so that *V*_60 Gy_ < 50%, *V*_65 Gy_ < 35%, and *V*_70 Gy_ < 25% (*V*_*x* Gy_: the percentage of the organ volume receiving *x* Gy or more). Similarly, the bladder exposure was limited so that *V*_65 Gy_ < 50% and *V*_70 Gy_ < 35% [17]. This study was approved by the Institutional Review Board (IRB) of the Gyeongsang National University Changwon Hospital (IRB 2017-08-012).

### 2.2. Body Contour Changes and Virtual Treatment Planning

**Figure 1:**
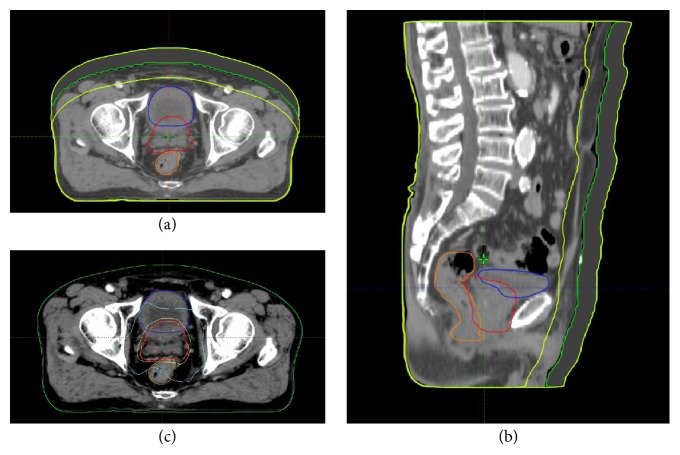
The changes in body contour and target contour. (a) Axial image and (b) sagittal image. The planning target volume (PTV) is shown in red, bladder in blue, and rectum in orange. (c) Dose distribution: the isodose lines are 100% (pink), 90% (yellow), and 50% (sky blue), respectively.

The body contours were artificially contracted and expanded in the conventional CT images from −2.0 cm to 2.0 cm, by 0.5 cm increments (Figure 1), to mimic the patients' WT changes. Bones and internal organs were kept in the same position. While the artificially expanded regions were assigned a CT density based on the surrounding soft tissue, the contracted region was replaced by the air density value. Nine different CT sets were prepared for each patient, which included the original CT scan, four expanded CT sets mimicking WT gain, and four contracted CT sets mimicking WT loss. For comparison, adaptive VMAT plans were made using these CT sets for each patient, while keeping all other planning parameters, such as the target volume, OAR, prescription dose, beam geometry, and dose-volume optimization criteria same as those in the original VMAT plan. The adaptive VMAT plans were made for three different photon energy changes (6-MV, 10-MV, and 15-MV). Consequently, a total of 27 comparable VMAT plans (on 9 different CT sets with 3 different energies) were made per patient.

### 2.3. Dosimetric Analysis

By analyzing the dose-volume histogram (DVH), each VMAT plan was compared in terms of PTV coverage, conformity index (CI), homogeneity index (HI), and doses to the rectum and bladder. PTV coverage was analyzed using the terms *D*_95%_ (*D*_*x*%_: the dose to *x*% of the volume) and *D*_max⁡_ for PTV. The recalculated plan was considered unacceptable, if the PTV *D*_95%_ is lower than 98% or the PTV *D*_max⁡_ is higher than 107% [18, 19]. The plan was also considered unacceptable, if the dose to the rectum and bladder exceeded the QUANTEC recommended dose constraints.

The conformity and homogeneity indices for all the plans were calculated using the following formula [20, 21]:

(1) $$\begin{array}{c} \text{Conformity index}=\frac{V_{RI}}{\text{TV}}, \end{array}$$

where *V*_RI_ is defined as the volume encompassed by the prescription isodose and TV is the target volume. The optimal value is 1 which corresponds to an ideal conformation. A CI larger than 1 indicates that the irradiated volume is greater than the target volume and includes some normal tissues, while CI lower than 1 indicates that only a part of the target volume is getting irradiated [20].

(2) $$\begin{array}{c} \text{Homogeneity index}=\frac{D_{5\%}}{D_{95\%}}. \end{array}$$

*D*_5%_ and *D*_95%_ are the minimum doses to 95% and 5% of the target volume, respectively. The ideal value is 1 and an increase of inhomogeneity results in an increased HI.

### 2.4. Gamma Index Analysis

Gamma index is a concept to calculate the difference between the calculated plan doses and the measured plan doses by specific quality assurance (QA) devices and is usually used in RT clinics for QA of IMRT and VMAT plans [22, 23]. However, in our study, we applied this concept to compare the two plans and to show the dose differences as a single numeric. A two-dimensional gamma index measured by the electronic portal imaging device (EPID) and calculated by the Varian portal dosimetry system (version 13.6) was used to quantitatively evaluate the changes in the actual delivered dose arising due to WT changes. We adopted the equation for gamma index suggested by Low et al. [23] and modified it as follows:

(3) $$\begin{array}{c} \text{Gamma index}=\min\left\{\;\Gamma \left(\;r_{\text{e}},r_{\text{s}}\;\right)\;\right\}\;\forall \left\{r_{\text{s}}\right\}, \end{array}$$

where *r*_e_ is a single measurement point in the experimental plan (with WT change) and *r*_s_ is a single measurement point in the standard plan (no WT change).

(4) Γre,rs=r2re,rsΔd2+δ2re,rsΔD2rre,rs=rs−re,δre,rs=Dsrs−Dere,

where Δ*d* and Δ*D* are the distance and dose passing criteria which were defined as 3 mm and 3% in this study, while *D*_*x*_ is the measured dose in the “s” (standard) or “e” (experimental) plans. The point is passed if the gamma value is 1 or below 1, and the point is failed if the gamma value is higher than 1. The gamma passing rate is defined as follows:

(5) Gamma passing rate=the number of passed points gamma<1the number of all points.

The experimental plan is regarded as unacceptable if the gamma passing rate is below 95% or the maximum gamma index is higher than 3.5.

**Figure 2:**
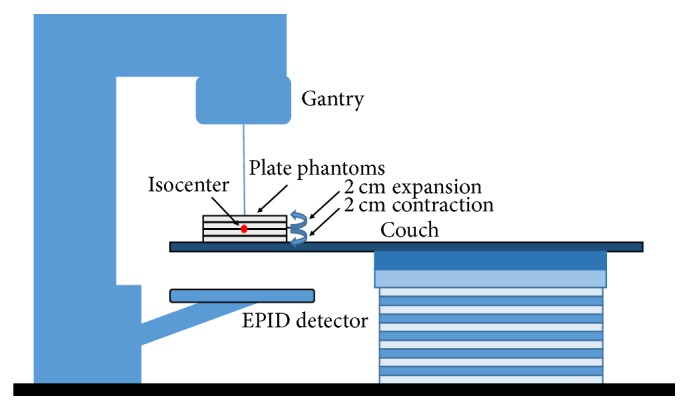
A simplified illustration to show the gamma index evaluation method.

**Figure 3:**
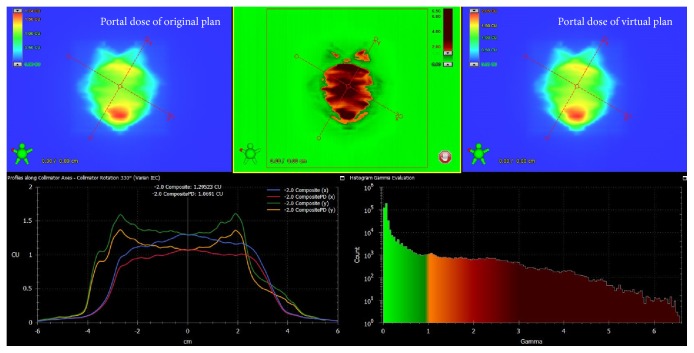
A gamma index analysis with the 3 mm/3% criterion for comparing the original plan and experimental plan using a −2.0 cm contracted body contour. The number of points falling beyond our gamma criteria is represented by the red spots in the middle image.

For this measurement, we used 0.5 and 1 cm thick plate phantoms to reflect the patients' body contour changes (−2.0 cm to 2.0 cm). A VMAT plan with the same monitor unit (MU) and gantry rotation was employed to irradiate these phantoms with varied thicknesses (Figure 2). The dose distribution measured using the 2 cm thick phantom was regarded as the standard (no WT change). This was compared to the dose distributions measured using phantoms with a thickness ranging from 0 to 4 cm. An example of the gamma index analysis result is shown in Figure 3.

## 3. Results

### 3.1. Target Coverage

**Figure 4:**
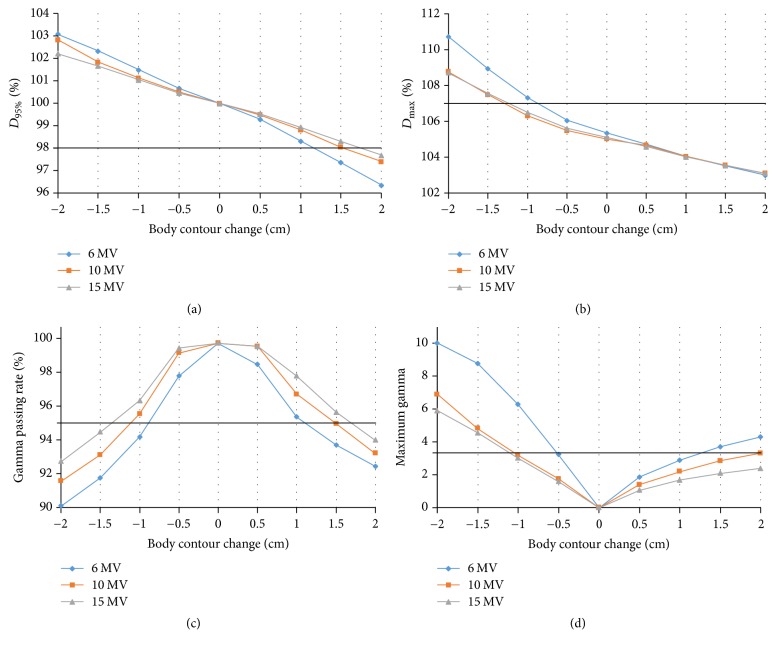
The changes in dosimetric parameters (a) *D*_95%_ and (b) *D*_max⁡_ for the PTV and the changes in (c) gamma passing rates and (d) maximum gamma values with changes in body contour for different photon energy levels. The solid lines are the acceptable threshold lines.

The results of the dosimetric analysis of target coverage are shown in Table 1 and Figures 4(a)-4(b). The mean *D*_95%_ for PTV decreased gradually as body contour expanded from −2.0 cm to 2 cm by 0.5 cm increments. Among the three different photon energy plans, the 6-MV plan showed the most prominent decrease. *D*_95%_ became lower than 98% when the body contours were expanded to 1.5 cm and 2.0 cm in the 6-MV and 10-MV plans, respectively. The mean *D*_max⁡_ for PTV increased as the body contour contracted and decreased as the body contour expanded. *D*_max⁡_ for PTV became higher than 107% when the body contour was contracted by 1.5 cm or more in the 10-MV plans and by 1.0 cm or more in the 6-MV plans. The mean CI also decreased gradually as the body contour expanded. The CI was lower than 0.5 when the body contour was expanded by 1.5 cm and 2.0 cm in the 6-MV and 10-MV plans, respectively. The mean HI for PTV deteriorated as the body contour was contracted or expanded. The results of the VMAT plans using 15-MV photon energy were similar to those using 10-MV photon energy.

### 3.2. Normal Tissue Sparing

**Figure 5:**
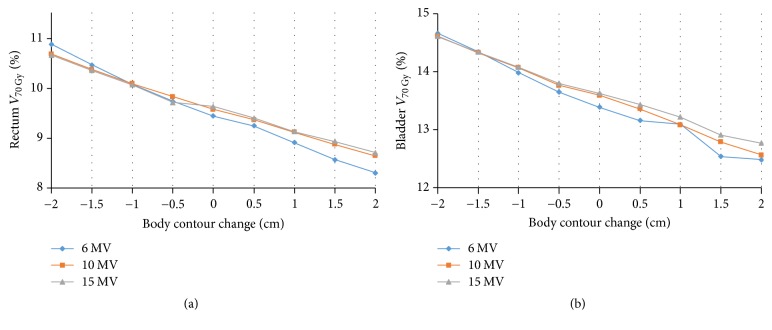
The changes in dosimetric parameters (a) rectum *V*_70 Gy_ and (b) bladder *V*_70 Gy_ with changes in body contour from −2.0 cm to 2.0 cm for different photon energy levels.

**Figure 6:**
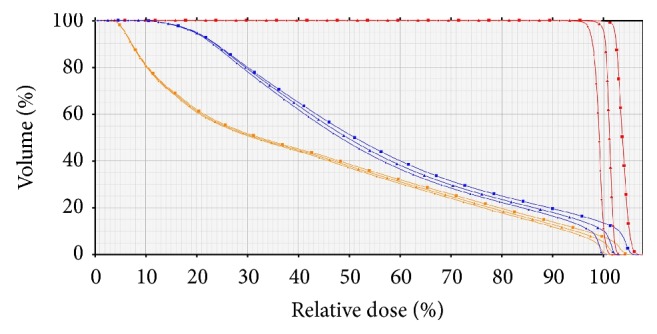
An example of a dose-volume histograms (DVH) of the volumetric modulated arc radiotherapy plan using 10-MV photon energy for a prostate cancer patient. The dose-volume histograms are indicated using the different colors (PTV, red; rectum, orange; bladder, blue) and types of lines (−2.0 cm body contour, square; original body contour, triangle; 2.0 cm body contour, small circle).

The dose to the rectum and bladder increased gradually as the body contour was contracted and decreased as the body contour was expanded. Among the three different photon energy plans, the 6-MV plan showed the most prominent decrease. The rectum *V*_70 Gy_ increased from 9.57% to 10.67% as the body contour was contracted by 2.0 cm in the 10-MV plans. The bladder *V*_70 Gy_ also increased from 13.59% to 14.61% in the 10-MV plans. However, these differences were relatively small and all parameters satisfied the normal tissue dose constraints even in the 6-MV plans with body contours contracted by 2.0 cm. Overall the detailed dose-volume data for *V*_70 Gy_ for the rectum and bladder are shown in Table 2 and Figure 5.  Figure 6 shows changes in the DVH of one patient.

### 3.3. Gamma Index Analysis

Table 3 and Figures 4(c)-4(d) show the changes in gamma passing rates and the maximum gamma index. The mean gamma passing rates decreased in both directions as the body contours were expanded or contracted. In the 6-MV plans, the gamma passing rates became lower than 95% both when the body contour was contracted by 1.0 cm or more and when the body contour was expanded by 1.5 cm or more. The maximum gamma index was also higher than 3.5 under the same conditions. In the 10-MV and 15-MV plans, the gamma index analysis showed unacceptable results when the body contour was either contracted by 1.5 cm or more or expanded by 2.0 cm or more.

## 4. Discussion

*What Can We Do If the Body Contour Change Impedes the Accuracy of Dose Delivery?* Since individual body contour changes cannot be accounted for by simple geometric correction methods, such as target position corrections by couch or patient shifts, other strategies should be considered. Some of these strategies include (a) using large PTV margins [24, 25] to cover various body contour changes, (b) tolerating the risk of over- or undertreating the target, or (c) replanning the treatment so as to adapt to the individual body changes [26, 27]. Ethically, ART is probably the most reasonable solution to date. Recently, Castelli et al. [28] have studied the benefits of adaptive replanning in head and neck cancers with frequent anatomical variations such as weight change and tumor shrinkage. They reported that weekly replanning could reduce the mean dose to the parotid glands by 5.1 Gy. This dose difference could reduce the absolute risk of xerostomia by an average of 11%. In our study on the prostate cancer, the adaptive replanning according to weight change had the benefits of increased *D*_95%_ of the target volume up to 2.61% and reduced *D*_max⁡_ of the target volume, rectum *V*_70 Gy_, and bladder *V*_70 Gy_ up to 3.76%, 1.1%, and 1.02%, respectively. However, despite this benefit there are no definite criteria when ART should be considered using body contour or WT changes.

*When Should ART Be Considered?* To the best of our knowledge, no studies have been conducted to address this question. In our study, as body contours changed (−2 cm to 2 cm), the dose delivered to the normal tissues did not cross the tolerance limit. However, the dosimetric parameters and gamma index analysis yielded consistent results for target coverage. With the use of 6-MV photon energy, the plan became unacceptable if the body contour was either contracted by 1 cm or more or expanded by 1.5 cm or more. However, with 10-MV or 15-MV photon energies, a contraction by 1.5 cm or expansion by 2.0 cm made the plan unacceptable. Therefore, ART should be considered if the depth of body contour becomes smaller than 1.5 cm or larger than 2.0 cm. Changes beyond these values could affect the target coverage and cause a failure in the gamma index analysis. The depth of body contour could be measured by acquiring in-room kV or MV cone-beam CT (CBCT) images or by simply measuring the source-to-skin distance (SSD) according to the equipment of each institution. However, when using only SSD, several factors should be considered, such as patient positioning and immobilization.

*What Is the Optimal Photon Energy for Prostate Cancer Patients?* Several studies have reported better dosimetric distribution with 10-MV plans compared to 6-MV plans for IMRT and VMAT [28, 29]. Plans with 15-MV have little advantage over the 10-MV plans but may carry an increased risk of neutron contamination [29, 30]. Our results demonstrate that the 10-MV plans are better than the 6-MV plans. The 10-MV plans resulted in smaller dose differences as the body contour or WT changed, compared to the 6-MV plans. However, since the differences between the 10-MV and 15-MV plans were relatively small and the risk of neutron contamination increased for the higher energy doses, to use the 10-MV photon beams for IMRT of prostate cancer is recommended.

*Limitations*. First, our study was limited to VMAT plans, treating only the prostate and seminal vesicles. Although the targets and OAR were contoured following the guidelines of RTOG, several planning factors such as the optimization skills could alter the study results. Therefore, our findings and the suggested cut-off values might not be applicable to all institutions. The second limitation is that, in the actual clinical cases, body contours do not change in a uniform manner as shown in Figure 1. In addition, the position of the internal organs could change as the body WT changes. However, these factors were not considered in this study. Further retrospective studies using WT changes from real clinical cases are needed to answer these questions. However, our study presents a guideline for the choosing the optimal time to give ART to prostate cancer patients with WT changes.

## 5. Conclusion

In conclusion, we have found that WT changes during prostate VMAT can cause considerable change in the target dose distribution. We suggest that when using 10-MV VMAT plans for patients with prostate cancer, the appropriate time to consider adaptive replanning is when the body contour becomes 1.5 cm smaller (WT loss) or 2.0 cm larger (WT gain) than the original value.

## Figures and Tables

**Table:** (a) Planning target volume coverage (%)

		Body contour changes (cm)								
		−2.0	−1.5	−1.0	−0.5	0	0.5	1.0	1.5	2.0
6-MV	*D*_95%_	103.08	102.33	101.49	100.66	100.00	99.28	98.31	*97.35*	*96.34*
	*D*_max⁡_	*110.74*	*108.94*	*107.34*	106.06	105.36	104.72	104.06	103.52	103.00
10-MV	*D*_95%_	102.83	101.83	101.13	100.51	100.00	99.48	98.81	98.04	*97.39*
	*D*_max⁡_	*108.78*	*107.50*	106.32	105.48	105.02	104.68	104.06	103.56	103.12
15-MV	*D*_95%_	102.22	101.66	101.05	100.46	100.00	99.52	98.93	98.30	*97.68*
	*D*_max⁡_	*108.72*	*107.56*	106.50	105.62	105.12	104.60	104.02	103.54	103.12

*D*_*x*%_: the percentage of the prescription dose covering *x*% of the volume. *Italic font: unacceptable*.

**Table:** (b) Conformity and homogeneity indices

		Body contour changes (cm)								
		−2.0	−1.5	−1.0	−0.5	0	0.5	1.0	1.5	2.0
6-MV	CI	1.18	1.14	1.10	1.05	1.00	0.91	0.62	0.33	0.19
	HI	1.047	1.037	1.032	1.029	1.028	1.029	1.033	1.037	1.043
10-MV	CI	1.15	1.11	1.08	1.03	1.00	0.93	0.74	0.50	0.31
	HI	1.040	1.039	1.029	1.028	1.027	1.028	1.030	1.033	1.036
15-MV	CI	1.14	1.11	1.07	1.02	0.99	0.94	0.78	0.55	0.39
	HI	1.042	1.037	1.033	1.031	1.030	1.026	1.031	1.033	1.034

CI, conformity index; HI, homogeneity index.

**Table 2:** The changes in dosimetric parameters for the rectum (R) and bladder (B) with the changes of body contour from −2.0 cm to 2.0 cm.

		Body contour changes (cm)								
		−2.0	−1.5	−1.0	−0.5	0	0.5	1.0	1.5	2.0
6-MV	R_*V*_70 Gy_	10.87	10.45	10.06	9.73	9.44	9.24	8.91	8.57	8.30
	B_*V*_70 Gy_	14.66	14.34	13.98	13.65	13.39	13.16	13.09	12.54	12.48
10-MV	R_*V*_70 Gy_	10.67	10.36	10.08	9.82	9.57	9.36	9.12	8.88	8.64
	B_*V*_70 Gy_	14.61	14.33	14.07	13.76	13.59	13.36	13.08	12.79	12.56
15-MV	R_*V*_70 Gy_	10.65	10.35	10.06	9.71	9.63	9.40	9.13	8.93	8.70
	B_*V*_70 Gy_	14.61	14.34	14.08	13.80	13.63	13.43	13.22	12.91	12.77

*V*_*x* Gy_, the percentage of the organ volume receiving *x* Gy or more; R_*V*_70 Gy_, rectum *V*_70 Gy_; B_*V*_70 Gy_, bladder *V*_70 Gy_.

**Table 3:** The changes in gamma index with changes in body contour from −2.0 cm to 2.0 cm.

		Body contour changes (cm)								
		−2.0	−1.5	−1.0	−0.5	0	0.5	1.0	1.5	2.0
6-MV	GPR	*90.10*	*91.80*	*94.30*	98.00	100.00	98.70	95.50	*93.80*	*92.50*
	MG	*10.00*	*8.76*	*6.27*	3.21	0.00	1.85	2.88	*3.69*	*4.29*
10-MV	GPR	*91.60*	*93.20*	95.70	99.40	100.00	99.80	96.90	95.10	*93.30*
	MG	*6.89*	*4.81*	3.19	1.75	0.00	1.39	2.19	2.85	3.30
15-MV	GPR	*92.80*	*94.60*	96.50	99.70	100.00	99.80	98.00	95.80	*94.10*
	MG	*5.90*	*4.55*	3.01	1.58	0.00	1.06	1.68	2.08	2.38

GPR, gamma pass rate; MG, maximum gamma values. *Italic font: unacceptable*.
